# Microstructure of the human metastatic vertebral body

**DOI:** 10.3389/fendo.2024.1508504

**Published:** 2025-01-06

**Authors:** Giulia Cavazzoni, Enrico Dall’Ara, Marco Palanca

**Affiliations:** ^1^ Department of Industrial Engineering, Alma Mater Studiorum - University of Bologna, Bologna, Italy; ^2^ Division of Clinical Medicine, The University of Sheffield, Sheffield, United Kingdom; ^3^ Insigneo Institute, The University of Sheffield, Sheffield, United Kingdom

**Keywords:** microstructural analysis, trabecular bone, vertebrae, spinal metastases, microCT

## Abstract

**Introduction:**

Bone spinal metastases disrupt the bone homeostasis, inducing a local imbalance in the bone formation and/or resorption, with consequent loss of the structural optimisation of the vertebrae and increase of the risk of fracture. Little is known about the microstructure of the metastatic tissue, the microstructure of the tissue surrounding the lesion, and how it does compare with vertebrae with no lesions observed on the biomedical images. A comprehensive assessment of the microstructural properties of the entire vertebral body can be obtained with micro computed tomography. In this study, we evaluated to what extent the vertebral body is affected by the presence of a metastatic lesion, the properties of the metastatic lesions, and whether the tissue surrounding the lesion has microstructural features similar to those of healthy tissue.

**Methods:**

A total of 30 metastatic vertebrae, including lytic (*N* = 12), blastic (*N* = 10), and mixed (*N* = 8) metastases, and 20 control vertebrae with no visible lesions on computed tomography were scanned using micro computed tomography (voxel size = 39 mm). The images were segmented and analysed to evaluate the microstructural properties in the entire vertebral body, in the lesion, and in the bone surrounding the lesion.

**Results:**

The microstructural properties evaluated on the entire vertebral bodies showed remarkable differences between metastatic and control vertebral bodies (p < 0.034) in terms of bone volume fraction, trabecular thickness, degree of anisotropy, connectivity density, and trabecular pattern factor. On the other hand, when the tissue surrounding the lesion was considered, no differences were found between metastatic and control vertebral bodies, except for differences in the degree of anisotropy (p = 0.008). All microstructural parameters measured in the regions including the lytic or the blastic metastases significantly differed (p < 0.001) from those in the tissues surrounding the lesions. The lytic lesions minimally affected the regions closest to the metastases, with significant differences only in the connectivity density. On the other hand, blastic metastases also affected the trabecular separation, the bone surface density, and the connectivity density in the closest tissue surrounding the lesion.

**Discussion:**

Most of the microstructural features of the trabecular bone in metastatic vertebrae were locally affected by lytic and blastic metastases, whereas the surrounding tissue showed a microstructure similar to that of adjacent vertebrae without visible lesions

## 1 Introduction

The bone tissue within the human vertebral body is a structure highly optimised to support daily loading, minimising the metabolic costs (1). The optimal bone mass adapts in size, shape, and microarchitecture to guarantee structural strength (2). However, some pathologies, such as primary and secondary tumours, disrupt the bone remodelling and cause imbalance in the bone homeostasis (3). In particular, bone metastases alter the healthy bone adaptation process, inducing lytic, blastic, or mixed lesions within the bone (4). Lytic metastases are characterised by local bone resorption due to the pathologic increased activity of the osteoclasts (3). Conversely, in the case of blastic metastases, the increased activity of osteoblasts leads the metastatic bone to appear as dense osteosclerotic lesions (3). Mixed metastases include both lytic and blastic lesions.

From a clinical point of view, bone metastases are characterised according to their radiologic appearances (i.e., type, size, and position) or through invasive analyses, such as histological assessment of bone biopsies (5). However, the radiation dose irradiated to the patient and the spatial resolution of clinical computed tomography (CT) images (typically with voxel size larger than 0.5 mm) are inadequate for accurately resolving three-dimensional (3D) bone features (6–8). Only the bone mineral density (BMD) is reliably evaluated by quantitative CT (qCT) and as a statistical description on relatively large volumes. Several studies compared the BMD measured by qCT with the bone volume fraction (BV/TV) measured by microCT and observed that they were well correlated (9–12). Thus, in addition to the clinical measurements, a deeper understanding of the bone tissue organisation (i.e., geometry, density, shape, and orientation of trabeculae) could be provided by *ex vivo* microstructural investigations. In fact, little is known about the microstructure of the metastatic tissue and the microstructure of the tissue surrounding the lesion with respect to the tissue in radiologically healthy vertebrae.

The microstructure of human vertebrae has been investigated using microCT imaging, which enabled the assessment of the trabecular bone volume fraction (BV/TV), trabecular thickness (Tb.Th.), trabecular separation (Tb.Sp.), and trabecular number (Tb.N.), among other standard microstructural parameters (13–15). Successful analyses have already been performed on bone cores (13, 16–25) and whole human vertebral bodies (26) without skeletal diseases, highlighting the heterogenous spatial distribution and arrangement of the trabeculae: BV/TV ranged from 6% to 36% (13, 16–24, 26), Tb.Th. ranged from 103 to 185 μm (13, 16–21, 23–25), Tb.Sp. ranged from 759 to 1,169 μm (13, 19, 20, 23, 24), Tb.N. ranged from 0.6 to 1.5 mm^−1^ (13, 16, 18–21, 23, 24), the connectivity density (Conn.D) ranged from 0.8 to 3.3 mm^−3^ (18, 19, 21, 23, 24, 26), the degree of anisotropy (DA) ranged from 1.42 to 2.23 (16, 18–21, 23), the structural model index (SMI) ranged from 1.79 to 2.66 (17–20, 23), and the trabecular pattern factor (Tb.Pf.) ranged from 2.00 to 5.65 mm^−1^ (16, 20). Agreements between the microstructural features and the mechanical properties [i.e., the vertebral strength (20, 26) and the risk of fracture (18, 19, 27, 28)] were also identified: the BV/TV in bone cores correlated well with the vertebral strength and failure (0.54 < *R* ^2^ < 0.77, *p* < 0.007) (19, 20), and this correlation was found to improve when measurements of local trabecular microstructural parameters were taken into account (0.85 < *R* ^2^ < 0.89, *p* < 0.0001). In addition, the SMI measured in the trabecular bone within the vertebral body was found to significantly negatively correlate with the failure load of the vertebral body (*R* = −0.76, *p* < 0.001) (19).

The microstructure in metastatic vertebrae has only been assessed in rat models (29, 30) or in human trabecular bone cores (7, 16, 21, 22). These studies highlighted the local structural changes that occur within the bone tissue affected by the metastases and suggested an increase of the risk of fracture of the metastatic vertebrae or the adjacent ones. In particular, the microstructure of human bone cores from vertebrae with lytic metastases had lower trabecular BV/TV (ranged between 5% and 25%) (7, 22, 31), Tb.Th. (between 100 and 250 μm) (7), Tb.N. (between 1 and 2 mm^−1^) (7), and Conn.D (between 2 and 50 mm^−3^) (7, 22) and higher Tb.Sp. (between 600 and 1,250 μm) (7) compared with human bone cores extracted from vertebrae with blastic metastases (7, 31) or vertebrae from donors without any skeletal diseases (22). On the other hand, bone cores from vertebrae with blastic metastases showed higher BV/TV (ranged between 20% and 60%) (7, 16, 21, 31), Tb.Th. (between 89 and 400 μm) (7, 16, 21), Tb.N. (between 2 and 7 mm^−1^) (7, 16, 21), Conn.D (between 50 and 250 mm^−3^) (7, 21), and Tb.Pf. (between −7 and 3 mm^−1^) (16) and lower Tb.Sp. (between 200 and 400 μm) (7) and DA (between 1.23 and 1.38) (16, 21) compared with bone cores extracted from healthy vertebrae from donors without skeletal diseases (16, 21, 22) and vertebrae with lytic metastases (7). Vertebrae with mixed metastases (7, 31) showed microstructural properties within the range of those of vertebrae with lytic and blastic lesions, and their strength was found to depend on the predominant type of tissue (30, 31).

The evidence provided by the analyses of bone cores is only partially representative of the entire microstructure of the vertebra, limiting the generalisation of the results. Assessing how the metastatic lesions affect the microstructure of the bone tissue in the whole vertebral body is crucial to better understand the effect of this skeletal disease on the structure and biomechanical competence of the metastatic vertebra. However, to date, this is still unknown.

The aim of the study was to provide a comprehensive assessment of the microstructural properties of the human vertebral bodies, with or without radiologically visible metastatic lesions.

## 2 Materials and methods

### 2.1 Sample and imaging

The study was approved by the Bioethics Committees of both the University of Bologna (reference no. 17325, 08/02/2019) and the University of Sheffield (reference no. 031782, 22/06/2020). The work was performed in accordance with the Declaration of Helsinki (1964, amended most recently in 2013).

A total of 15 spines from human donors (7 men and 8 women) (**Table 1**) with medical history of spinal metastases spread from different types of primary tumours were obtained from an ethically approved donation program (Anatomy Gift Registry, AGR). The spines were selected from non-osteoporotic donors without any signs of fracture and without any history of spinal surgery and spinal fixation. Each spine was scanned using qCT (AquilionOne, Canon Medical Systems Corporation (Toshiba Medical Systems Corporation), Ōtawara, Tochigi, Japan) following an optimised bone protocol (voltage, 120 kVp; current, 200 mA; slice thickness, 1 mm; in-plane resolution, approximately 0.45 mm) in order to identify the metastatic vertebrae and the type of metastasis. A total of 27 thoracolumbar specimens consisting of a metastatic vertebra and an adjacent control vertebra were extracted from the spines. The vertebrae were therefore assigned to two groups of specimens (i.e., metastatic and control vertebrae) nearly balanced in terms of vertebral level, sex, and age. The soft tissues and the posterior elements were removed, each specimen was wrapped with gauzes soaked in saline solution, and then scanned using microCT (VivaCT80, Scanco Medical, Bruttisellen, Switzerland) within a radiotransparent custom-built jig (32, 33). In four cases, a vertebral body did not fit the microCT holder; thus, the single vertebra was removed from the study. Imaging was performed aligning the cranial–caudal axis of the vertebra to the axis of the scanner and using parameters previously optimised for scanning the whole vertebral body (32, 34): current, 114 mA; voltage, 70 kVp; integration time, 300 ms; power, 8 W; 750 projections/180°C; isotropic voxel size, 39 μm. The standard reconstruction algorithm recommended by the manufacturer was used with a polynomial correction based on scans of a wedge phantom with 1,200 mg/cm^3^ of hydroxyapatite (HA) to reduce the beam hardening artefact (35). MicroCT images were used to amend the primary evaluation based on the clinical CT images by confirming the presence and the type of metastasis for each vertebra. After updating the classification of the metastasis type (36), the sample included 30 metastatic vertebrae with lytic (*N* = 12 from six donors), blastic (*N* = 10 from five donors), and mixed (*N* = 8 from four donors) metastases and 20 control vertebrae from 10 donors (**Table 1**). The sample included vertebrae from the upper thoracic spine (T3–T8, *N* = 13 for metastatic vertebrae and *N* = 10 for control vertebrae), the lower thoracic spine (T9–T12, *N* = 6 for metastatic vertebrae and *N* = 6 for control vertebrae), or the lumbar spine (L1–L5, *N* = 11 for metastatic vertebrae and *N* = 4 for control vertebrae) (details in **Table 1**).

**Table 1:** Details of the donors for the metastatic and control (without any radiological signs of metastatic lesions identifiable from clinical CT) vertebrae.

Vertebra ID	Donor	Type of primary tumour	Age (years)	Gender	Spine level	Group	Metastasis size (% of total vertebral body volume)
1	A	Adrenal	81	M	T4	Lytic	30
2					T6	Lytic	3
3	B	Lung	51	F	T3	Lytic	20
4					L2	Lytic	43
5	C	Breast	82	F	T6	Lytic	4
6					T11	Lytic	6
7					L4	Lytic	16
8	D	Breast	46	F	T4	Lytic	25
9					T5	Lytic	3
10					T12	Lytic	49
11	E	Adenocarcinoma	62	F	T5	Lytic	9
12	F	Nasopharyngeal	72	M	T6	Lytic	6
13	A	Adrenal	81	M	L1	Blastic	12
14	G	Bladder	75	M	T12	Blastic	3
15	H	Prostate	66	M	L2	Blastic	100
16	I	Prostate	78	M	L1	Blastic	35
17					L2	Blastic	54
18					L4	Blastic	75
19					L5	Blastic	56
20	J	Lung	73	F	T7	Blastic	45
21					T12	Blastic	13
22					L2	Blastic	100
23	K	Breast	55	F	T8	Mixed	30
24					T11	Mixed	100
25	L	Prostate	83	M	L3	Mixed	45
26					L4	Mixed	71
27	M	Breast	51	F	T6	Mixed	15
28					T12	Mixed	3
29	N	Prostate	52	M	T7	Mixed	100
30					T8	Mixed	52
31	A	Adrenal	81	M	T3	Control	–
32	B	Lung	51	F	T4	Control	–
33					L3	Control	–
34	C	Breast	82	F	T7	Control	–
35					T10	Control	–
36					L3	Control	–
37	E	Adenocarcinoma	62	F	T6	Control	–
38	F	Nasopharyngeal	72	M	T7	Control	–
39	G	Bladder	75	M	T11	Control	–
40	J	Lung	73	F	T8	Control	–
41					T11	Control	–
42					L3	Control	–
43	K	Breast	55	F	T7	Control	–
44					T10	Control	–
45	M	Breast	51	F	T7	Control	–
46					L1	Control	–
47	O	Uterine	59	F	T7	Control	–
48					T8	Control	–
49					T11	Control	–
50					T12	Control	–

### 2.2 Microstructure assessment

The microCT images were processed to assess the microstructure of the vertebral bodies (15, 16, 21, 37). A 3D median filter (isotropic support equal to 0.5) was applied to reduce the high-frequency noise of the microCT images without reducing the contrast between the bone and the marrow (14, 15). A single level threshold, calculated as the value identified by the Otsu thresholding algorithm (ImageJ, National Institute of Health, Bethesda, MD, USA) increased by 5% (38), was applied to segment the images. This threshold value was determined from a preliminary analysis where corrections of ±5% or ±10% of the automatically calculated Otsu threshold value were explored. The +5% correction was found to be the optimal threshold value that best preserved the trabecular structure after visual inspection (38).

Despite the fact that the analysed mineralised tissue included both trabecular bone and blastic metastatic tissue, the nomenclature for the standard trabecular 3D microstructural parameters widely defined in the literature was used (15, 39, 40). The following 3D microstructural parameters (15, 25, 41, 42) were calculated using CTAnalyzer (V1.17.7.2, Bruker, MA, USA): BV/TV (in percent), Tb.Th. (in micrometres), standard deviation of the trabecular thickness (SD_Tb.Th., in micrometres), Tb.Sp. (in micrometres), Tb.N. (1/mm), DA, Conn.D (mm^-3^), bone surface density (BS/TV, 1/mm), SMI, and Tb.Pf.

Three volumes of interest (VOIs) were defined. All segmentations were performed by the same expert operator (GC; Amira 6.2, Thermo Fisher Scientific, Waltham, MA, USA).

**Figure 1:**
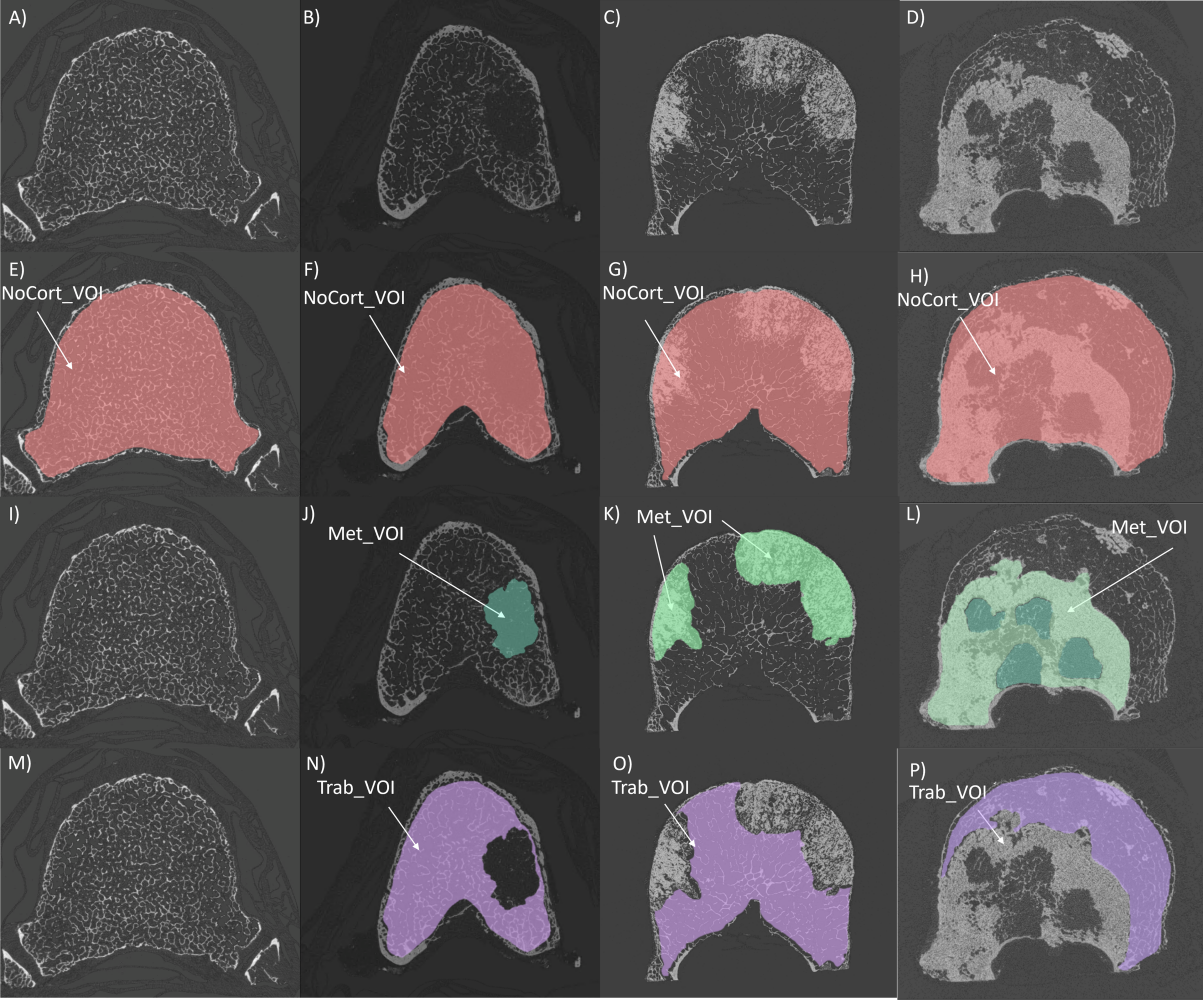
First line: microCT cross-sections of a control vertebra **(A)**, a vertebra with lytic **(B)**, blastic **(C)** and mixed **(D)** metastases. Second line: microCT cross-sections with overlapped masks representing the NoCort_VOI in red (E, F, G, H), which excludes the cortical shell and the endplates and include the metastatic lesion. Third line: microCT cross-section of a control vertebra **(I)**, a vertebra with lytic **(J)**, blastic **(K)**, and mixed **(L)** metastases with overlapped masks representing the Met_VOI in dark green for lytic lesions and light green for blastic lesions. Fourth line: microCT cross-sections of a control vertebra **(M)**, a vertebra with lytic **(N)**, blastic **(O)**, and mixed **(P)** metastases with overlapped masks representing the Trab_VOI in purple, which excludes both the cortical shell and the metastatic lesion, only for metastatic vertebrae

NoCort_VOI was defined as the volume of the vertebral body excluding the cortical shell and endplates (**Figures 1A–D**). The contours were manually defined every 20 slices and interpolated using a trilinear interpolation (**Figures 1E–H**).

Met_VOI was defined as the volume of the metastatic lesion(s) within the NoCort_VOI (**Figures 1J–L**). Since a proper automatic segmentation tool for bone metastases or even a gold standard procedure for tumour boundary definition is not available (43), the Met_VOI was obtained by manually segmenting the volume of the metastatic lesion(s). Manual segmentation was chosen due to the heterogeneity of the radiological appearance of the lesions making a single level threshold not adequate for segmentation (31, 44). Moreover, benign features such as fractures, blood vessels, bone islands, osteophytes, and degenerative changes could create further inaccuracies when using automatic segmentation (44). The contours of the metastatic tissue were defined every 20 microCT slices, and the volume of the lesion was created by applying a trilinear interpolation. The size of the metastases was expressed as a percentage of the total volume of the vertebral body (i.e., NoCort_VOI) (**Table 1**). It should be noted that lytic lesions in the early formation stages are characterised by a reduction of the number of trabeculae, which is followed by an increase of the focal bone resorption (4, 7, 31, 45). In particular, in this study, the borders of the lesions were usually clearly distinguishable from the surrounding tissue, and there was some mineralised tissue within the lytic lesions unconnected from its border. Therefore, the density within the lytic tissue was assessed.

Trab_VOI was defined only for metastatic vertebrae and consisted of the volume of the vertebral body excluding both the cortical shell (i.e., NoCort_VOI) and the metastatic lesion(s) (i.e., Met_VOI). Specifically, Trab_VOI was created by subtracting the Met_VOI from the NoCort_VOI (**Figures 1N–P**).

**Figure 2:**
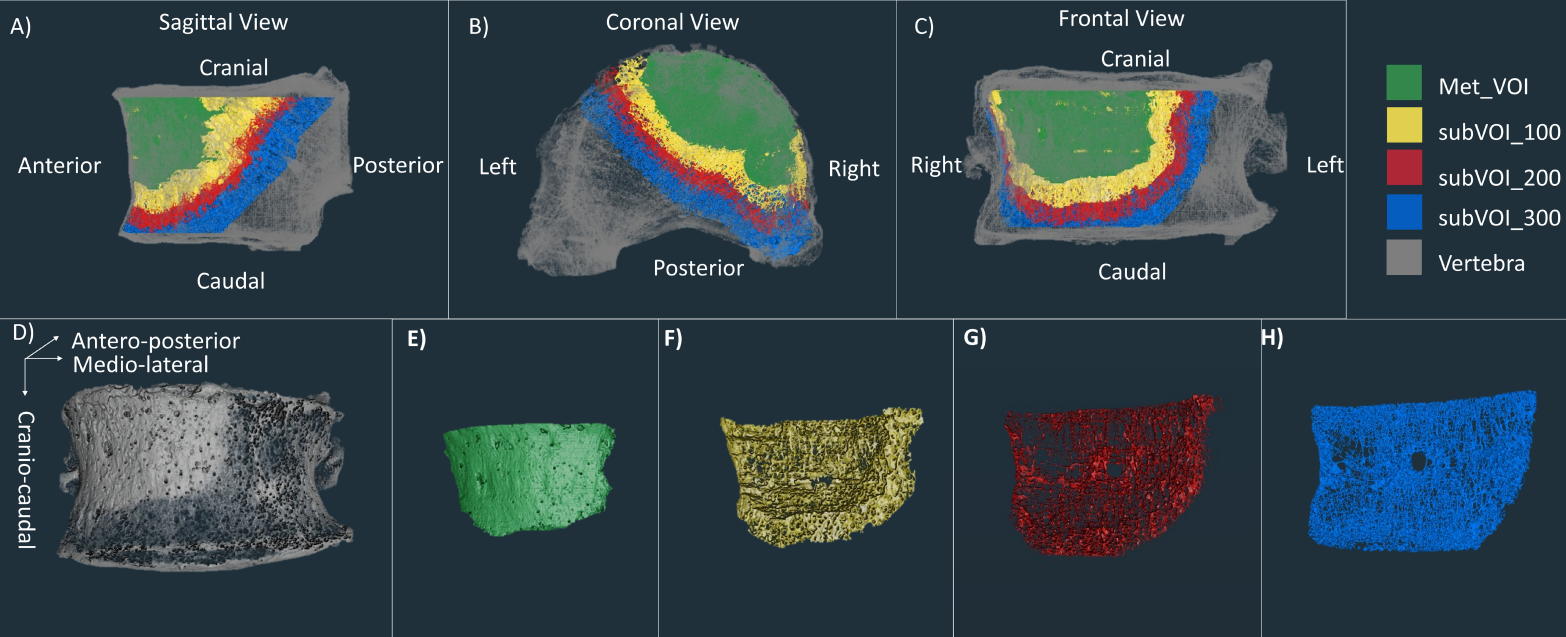
*First line*: microCT of a vertebra with blastic metastases (*grey*) with overlapped microCT of the Met_VOI (*green*), subVOI_100 (*yellow*), subVOI_200 (*red*), and subVOI_300 (*blue*) in the sagittal **(A)**, coronal **(B)**, and frontal **(C)** planes. *Second line*: microCT of the vertebra with blastic metastases (*grey*) **(D)**, Met_VOI (*green*) **(E)**, subVOI_100 (*yellow*) **(F)**, subVOI_200 (*red*) **(G)**, and subVOI_300 (*blue*) **(H)**.

In order to perform microstructural analysis of the regions surrounding the metastatic lesion, three subVOIs were created using ImageJ (**Figure 2**). These subVOIs had a thickness of 100 voxels, containing therefore at least three intertrabecular lengths, fundamental to obtaining reliable morphometric measurements (15). SubVOI_100 (**Figure 2F**) was defined between an internal surface defined by the Met_VOI external surface and an external surface defined by three-dimensionally dilating the Met_VOI external surface of 100 voxels (3.9 mm). SubVOI_200 (**Figure 2G**) was defined between the external surface of subVOI_100 and the external surface of subVOI_100 three-dimensionally dilated of 100 voxels. SubVOI_300 (**Figure 2H**) was defined between the external surface of subVOI_200 and the external surface of subVOI_200 three-dimensionally dilated of 100 voxels. These three subVOIs were defined only for vertebrae with lytic and blastic metastases as the multiple lesions in vertebrae with mixed metastases reduce the continuity and extension of the subregions. The DA and SMI were not calculated for the subVOIs due to potential issues associated with the calculation of these parameters on irregular and relatively thin regions of interest.

### 2.3 Statistical analysis

All statistical analyses were performed in Jamovi (46), with a significance level equal to 0.05.

Normal distribution of the volumes of the metastases was assessed using the Shapiro–Wilk test. When data were normally distributed, homoscedasticity was tested using Levene’s test. In order to evaluate whether the different types of lesions (i.e., lytic, blastic, and mixed) have different extensions, the volumes of the lesions were compared using Welch’s ANOVA with the Games–Howell *post-hoc* test.

The selection of multiple vertebrae per spine can introduce clustering (non-independence) of the data. Therefore, a linear mixed-effects model (LMM) was fitted to examine the effect of specimens from the same donors (random effect) on the following:

- Volumes of the lytic and blastic lesions (fixed effect) in vertebrae with mixed metastases;
- 3D microstructural parameters (fixed effect) in the overall volume of the vertebral body excluding the cortical shell (NoCort_VOI): i) between the metastatic (data pooled for vertebrae with lytic, blastic, and mixed metastases) and the control vertebrae and ii) among the four different groups of vertebrae (control vertebrae and vertebrae with lytic, blastic, and mixed metastases). *Post-hoc* analysis (Fisher’s least significant difference, LSD) was performed to compare the vertebrae with lytic, blastic, or mixed metastases and the control vertebrae.
- 3D microstructural parameters (fixed effect) in the trabecular bone surrounding the metastases (Trab_VOI): i) between the metastatic (data pooled for vertebrae with lytic, blastic, and mixed metastases) and the control vertebrae and ii) among the four different groups of vertebrae (control vertebrae and vertebrae with lytic, blastic, and mixed metastases). *Post-hoc* analysis (Fisher’s LSD) was performed to compare the vertebrae with lytic, blastic, or mixed metastases and the control vertebrae.
- 3D microstructural parameters (fixed effect) among the Met_VOIs, the different subVOIs (subVOI_100, subVOI_200, and subVOI_300), and the control vertebrae. *Post-hoc* analysis (Fisher’s LSD) was performed to compare the groups. This model was fitted for vertebrae with lytic and blastic metastases separately.

The LMM reported *F*-test significance (*F*) and *p*-value significance (*p*) to assess the significance of the fixed effect and the intraclass correlation coefficient (ICC) to assess the impact of the random effect.

## 3 Results

Trab_VOI was defined only for 26 of 30 metastatic vertebrae due to the vertebral bodies of four vertebrae being completely occupied by blastic (specimen IDs 15 and 22) or mixed (specimen IDs 24 and 29) metastases. In these blastic specimens (specimen IDs 15 and 22), it was not possible to define the subVOIs. Moreover, it was not possible to define the subVOI_200 for specimen ID 18 and the subVOI_300 for specimen IDs 18 and 19 as they fell outside the boundary of the volume of NoCort_VOI due to the large size of the metastatic lesion (75% and 56% of the vertebral body volume for specimen IDs 18 and 19, respectively).

The volumes of the lesions were significantly different among the vertebrae with lytic, blastic, or mixed metastases (*p* = 0.018), although no specific differences were observed in the *post-hoc* analysis. The size of the lytic metastases ranged between 3% and 49% (average, 18% of the volume of the vertebral body; volume range, between 0.2 and 10.3 cm^3^), that of the blastic metastases ranged between 3% and 75% (average, 37% of the volume of the vertebral body; volume range, between 0.7 and 31.0 cm^3^), and that of the mixed metastases ranged between 3% and 71% (average, 36% of the volume of the vertebral body; volume range, between 0.6 and 19.6 cm^3^) (**Table 1**). In the case of mixed metastases, the volume of the blastic lesions was significantly higher than that of the lytic lesions (+458%; *F* = 12.9, *p* = 0.016, ICC = 0.623).

The descriptive statistics of the 3D microstructural parameters in NoCort_VOI and in Trab_VOI for each group are shown in **Table 2**. See **Supplementary Table 1** for the individual values for each specimen.

**Table 2:** Three-dimensional microstructural parameters measured in the metastatic and control vertebral bodies, in NoCort_VOI and Trab_VOI, reported as the median and interquartile range (IQR) for each group.

		Control	Lytic				Blastic				Mixed			
		NoCort_VOI	NoCort_VOI	*p*	Trab_VOI	*p*	NoCort_VOI	*p*	Trab_VOI	*p*	NoCort_VOI	*p*	Trab_VOI	*p*
BV/TV (%)	Median	11	11	NS	13	NS	28	<0.001	12	NS	35	0.011	23	0.005
	(IQR)	(9–14)	(9–13)		(10–16)		(16–52)		(10–22)		(21–43)		(13–28)	
	%diff		+3%		+25%		+161%		+14%		+230%		+112%	
Tb.Th. (μm)	Median	165	182	NS	185	NS	209	<0.001	171	NS	301	<0.001	235	<0.001
	(IQR)	(157–181)	(163–196)		(161–193)		(196–283)		(164–189)		(252–324)		(205–263)	
	%diff		+10%		+12%		+27%		+4%		+82%		+42%	
SD_Tb.Th. (μm)	Median	79	94	NS	93	NS	107	0.018	86	NS	151	<0.001	120	<0.001
	(IQR)	(68–99)	(85–100)		(79–98)		(89–135)		(78–103)		(128–161)		(110–145)	
	%diff		+19%		+18%		+36%		+9%		+91%		+53%	
Tb.Sp. (μm)	Median	988	999	0.046	824	NS	788	NS	912	NS	724	NS	746	NS
	(IQR)	(837–1,071)	(894–1,261)		(744–873)		(369–1,050)		(599–1,049)		(566–956)		(576–875)	
	%diff		+1%		−17%		−20%		−8%		−27%		−24%	
Tb.N. (1/mm)	Median	0.65	0.63	NS	0.79	NS	1.29	0.003	0.69	NS	1.08	NS	0.95	NS
	(IQR)	(0.53–0.77)	(0.51–0.73)		(0.65–0.82)		(0.69–1.82)		(0.59–1.23)		(0.88–1.42)		(0.68–1.01)	
	%diff		−3%		+21%		+99%		+7%		+67%		+47%	
BS/TV (1/mm)	Median	2.47	2.5	NS	3.03	NS	5.1	<0.001	2.76	NS	3.71	NS	3.67	NS
	(IQR)	(2.05–2.84)	(2.04–2.86)		(2.53–3.25)		(2.81–6.25)		(2.31–5.22)		(3.47–5.24)		(2.91–4.14)	
	%diff		+1%		+23%		+107%		+12%		+50%		+49%	
DA	Median	1.4	1.35	NS	1.36	NS	1.22	0.003	1.22	0.003	1.2	<0.001	1.16	0.007
	(IQR)	(1.33–1.45)	(1.21–1.41)		(1.18–1.41)		(1.12–1.28)		(1.19–1.28)		(1.12–1.25)		(1.14–1.28)	
	%diff		−3%		−3%		−13%		−12%		−14%		−17%	
Conn.D(mm^-3^)	Median	6	7	NS	8	NS	27	<0.001	9	NS	9	NS	12	NS
	(IQR)	(4–7)	(6–8)		(6–9)		(9–41)		(6–28)		(5–20)		(7–20)	
	%diff		+21%		+34%		+378%		+53%		+62%		+112%	
SMI	Median	1.7	1.8	NS	1.8	NS	0.7	NS	1.8	NS	0.6	NS	1.9	NS
	(IQR)	(1.4–1.8)	(1.7–2.0)		(1.6–1.9)		(−0.7 to 1.7)		(1.6–2.1)		(−1.4 to 1.2)		(1.0–2.3)	
	%diff		+4%		+3%		−61%		+7%		−66%		+12%	
Tb.Pf. (1/mm)	Median	5.1	5	NS	4.8	NS	−2.3	<0.001	5.1	NS	−2	NS	4.2	NS
	(IQR)	(3.1–5.6)	(4.1–6.3)		(3.6–6.2)		(−9.1 to 1.0)		(3.7–6.5)		(−6.2 to 0.0)		(1.1–5.7)	
	%diff		−3%		−7%		−144%		−1%		−138%		−19%	

Percentage difference (%diff) was evaluated with respect to the control vertebrae and *p*-values are reported. Significance level, 0.05. “NS” represents not significant differences.

*BV/TV*, bone volume fraction; *Tb.Th.*, trabecular thickness; *SD_Tb.Th.*, standard deviation of the trabecular thickness; *Tb.Sp.*, trabecular separation; *Tb.N.*, trabecular number; *BS/TV*, bone surface density; *DA*, degree of anisotropy; *Conn.D*, connectivity density; *SMI*, structural model index; *Tb.Pf.*, trabecular pattern factor.

### 3.1 Microstructural analysis in NoCort_VOI

The BV/TV (+68%; *F* = 6.06, *p* = 0.019, ICC 0.779), Tb.Th. (+22%; *F* = 10.3, *p* = 0.003, ICC = 0.570), SD_Tb.Th. (+34%; *F* = 9.55, *p* = 0.004, ICC = 0.512), and Conn.D (+49%; *F* = 4.88, *p* = 0.034, ICC = 0.699) were significantly higher in the metastatic group than in controls. Conversely, the DA (−14%; *F* = 15, *p* = 0.001, ICC = 0.235) and Tb.Pf. (−92%; *F* = 5.13, *p* = 0.03, ICC = 0.904) were significantly lower in the metastatic group than in controls. The other 3D microstructural parameters were not significantly different between the metastatic and control vertebrae.

**Figure 3:**
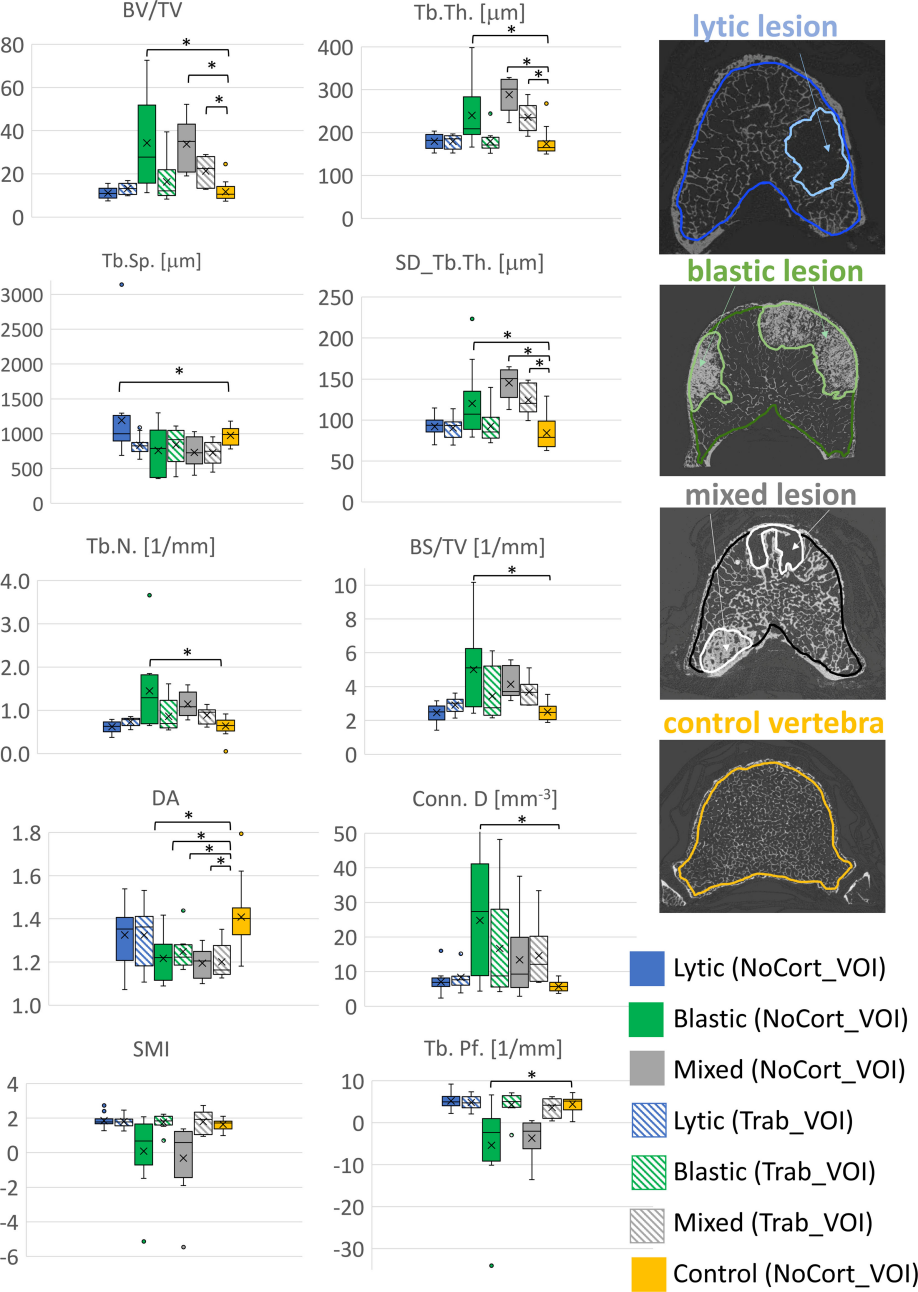
Boxplot of the 3D microstructural parameters of vertebral bodies with lytic (blue), blastic (green), mixed (grey) metastases and of control vertebral bodies (yellow) reported in solid colour for the NoCort_VOI and stripes colour for the Trab_VOI. The box is limited by the first and the third quartile. Whiskers represent the lowest and highest data point in the data set excluding any outliers, which are reported as open circles. Mean and median values over the group are represented by a cross and a horizontal line, respectively. Statistically significant differences between the metastatic groups and control group are highlighted with *.

The type of vertebra (i.e., controls or with lytic, blastic, or mixed metastases) was a significant factor for most of the morphometric parameters (0.001 < *p* < 0.043), except for SMI (*p* = 0.064) (**Table 2**, **Figure 3**). The *post-hoc* test revealed that vertebral bodies with blastic and mixed metastases showed larger (*p* < 0.018) values of BV/TV, Tb.Th., and SD_Tb.Th and smaller values of DA compared with the control vertebral bodies (**Table 2**, **Figure 3**). The vertebral body with blastic metastases, in addition, showed larger (*p* < 0.001) values of Tb.N. and Conn.D and smaller (*p* < 0.001) values of Tb.Pf. than the control vertebral bodies. No significant differences were found between the vertebral bodies with lytic metastases and the control vertebral bodies, except for larger Tb.Sp. (*p* = 0.046).

### 3.2 Microstructural analysis in Trab_VOI

No significant differences were found between the microstructural parameters measured in the trabecular bone surrounding the metastatic lesions and those measured in the trabecular bone of the control vertebrae (NoCort_VOI), except for DA (−17%; *F* = 7.74, *p* = 0.008, ICC = 0.364).

Among the different types of vertebrae (i.e., lytic, blastic, mixed, and control), only BV/TV (*F* = 3.03, *p* = 0.0042, ICC = 0.217), Tb.Th. (*F* = 5.83, *p* = 0.002, ICC = 0.295), SD_Tb.Th. (*F* = 5.58, *p* = 0.003, ICC = 0.169), and DA (*F* = 3.82, *p* = 0.017, ICC = 0.416) (**Table 2**, **Figure 3**) were significantly different. In particular, the *post-hoc* test revealed that the DA evaluated in the trabecular bone surrounding the blastic and mixed lesions was lower (*p* = 0.033 and *p* = 0.009, respectively) than that in the control vertebrae (**Table 2**, **Figure 3**). Moreover, the trabecular bone surrounding the mixed metastases showed larger (*p* < 0.005) values of BV/TV, Tb.Th., and SD_Tb.Th. than the trabecular bone of the control vertebrae.

### 3.3 Microstructural analysis in the regions surrounding the metastases

**Figure 4:**
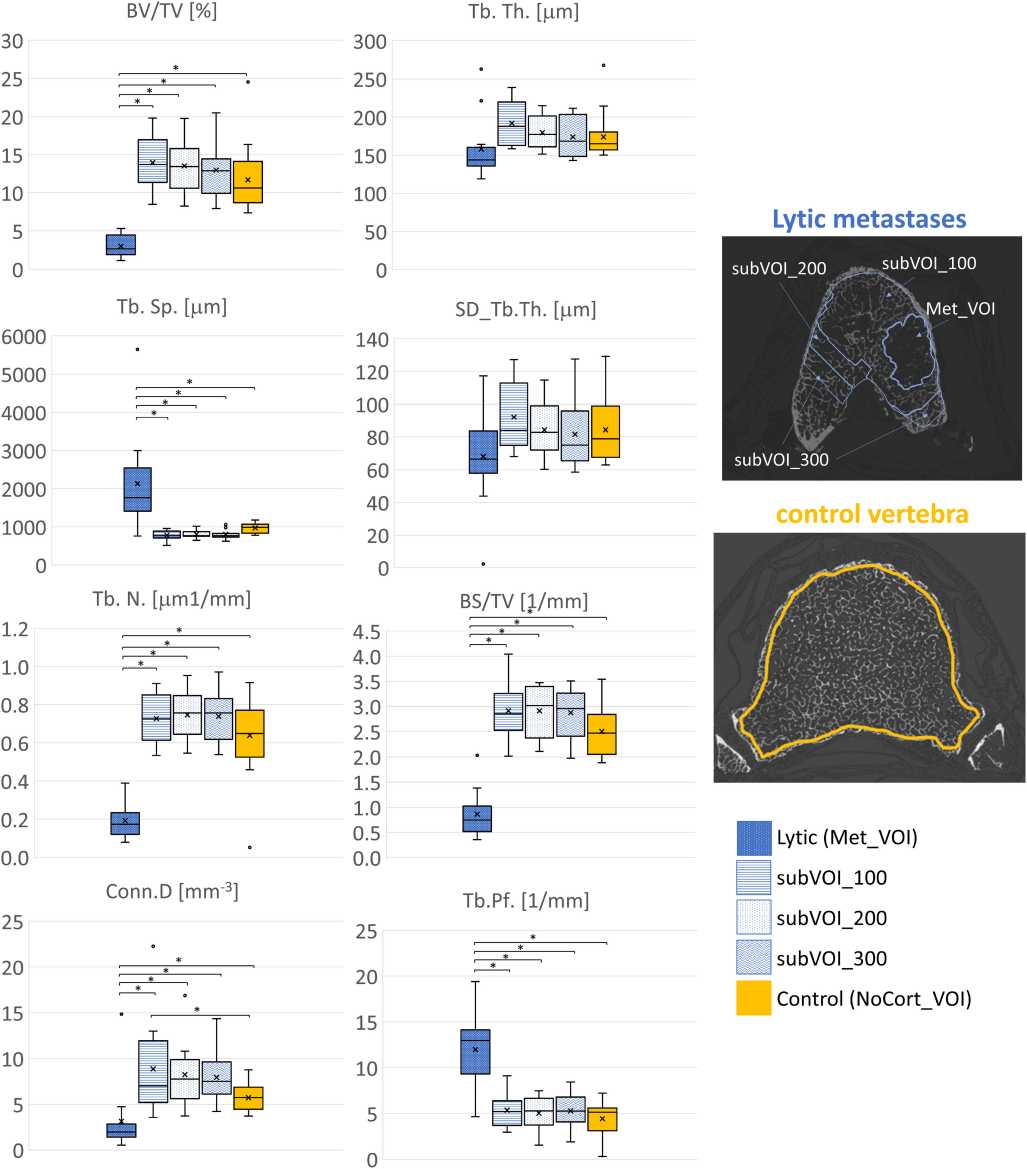
Box plot of the 3D microstructural parameters of the lytic metastases (*blue*, *dots dark background*), of the trabecular bone in subVOI_100 (*blue*, *horizontal line*), subVOI_200 (*blue*, *dots white background*), and subVOI_300 (*blue*, *zigzag line*), and the control vertebrae (*yellow*) in NoCort_VOI. The *box* is limited by the first and the third quartile. *Whiskers* represent the lowest and highest data points in the dataset excluding any outliers. The mean and median values over the group are represented by a *cross* and a *horizontal line*, respectively. Statistically significant differences between the metastatic groups and control group are highlighted with an *asterisk*.

In vertebrae with lytic metastases, most of the microstructural parameters resulted significantly different among the subVOIs and the Met_VOI: BV/TV (*F* = 42.9, *p* < 0.001, ICC = 0.635), Tb.Sp. (*F* = 16.0, *p* < 0.001, ICC = 0.109), Tb.N. (*F* = 71.4, *p* < 0.001, ICC = 0.657), BS/TV (*F* = 80.1, *p* < 0.001, ICC = 0.563), Conn.D (*F* = 6.48, *p* < 0.001, ICC = 0.209), and Tb.Pf. (*F* = 25.1, *p* < 0.001, ICC = 0.229). The lytic metastases (Met_VOI) (**Table 3**, **Figure 4**) showed values of BV/TV, Tb.N., BS/TV, and Conn.D lower (*p* < 0.001) than those measured in the subVOIs and the control vertebrae and values of Tb.Sp. and Tb.Pf. higher (*p* < 0.001) than those measured in the subVOIs and the control vertebrae. Moreover, the 3D microstructural parameters measured in the three subVOIs of the vertebral bodies with lytic metastases were similar (*p* > 0.219) and were not different from those measured in the control vertebrae (*p* > 0.054). However, the Conn.D measured in subVOI_100 for the vertebrae with lytic metastases differed from that measured in the control vertebrae (+22%; *p* = 0.032) (**Figure 4**).

**Table 3:** Three-dimensional microstructural parameters measured in the vertebrae with lytic and blastic metastases in Met_VOI, subVOI_100, subVOI_200, and subVOI_300 and in the control vertebrae in NoCort_VOI.

		Lytic				Blastic				Control
		Met_VOI	subVOI_100	subVOI_200	subVOI_300	Met_VOI	subVOI_100	subVOI_200	subVOI_300	NoCort_VOI
BV/TV (%)	Median (IQR) %diff	3 (2–4)	14^a^ (11–17) +419%	13^a^ (11–16) +408%	13^a^ (10–14) 387%	47 (31–66)	15^b^ (9–22) −68%	11^b^ (7–13) −77%	9^b^ (7–12) −80	11^a,b^ (9–14) +302%, −77%
Tb.Th. (μm)	Median (IQR) %diff	144 (136–160)	188 (263–220) NS	177 (161–201) NS	168 (148–203) NS	258 (217–287)	176^b^ (164–190) −32%	160^b^ (148–168) −38%	149^b^ (146–173) −42%	165^b^ (157–181) NS, −36%
SD_Tb.Th. (μm)	Median (IQR) %diff	66 (58–84)	84 (75–113) NS	83 (72–99) NS	75 (65–96) NS	128 (99–139)	88^b^ (75–99) −31%	75^b^ (63–94) −42%	70^b^ (65–79) −46%	79^b^ (68–99) NS, −38%
Tb.Sp. (μm)	Median (IQR) %diff	1765 (1,410–2,544)	768^a^ (708–890) −56%	770^a^ (755–872) −56%	769^a^ (731–828) −56%	305 (253–473)	822^b^ (584–939) +170%	977^b^ (759–1,024) +221%	907^b^ (839–1,005) 198%	988^a,b^ (837–1,071) −44%, +224%
Tb.N. (1/mm)	Median (IQR) %diff	0.17 (0.12–0.23)	0.72^a^ (0.61–0.85) +320%	0.76^a^ (0.64–0.85) +338%	0.76^a^ (0.62–0.83) +338%	1.90 (1.1–2.3)	0.77^b^ (0.61–1.27) −59%	0.61^b^ (0.45–0.77) −68%	0.59^b^ (0.49–0.69) −69%	0.65^a,b^ (0.53–0.77) +276%, −66%
BS/TV (1/mm)	Median (IQR) %diff	0.75 (0.51–1.02)	2.85^a^ (2.53–3.26) +283%	3.02^a^ (2.37–3.39) +305%	2.96^a^ (2.41–3.26) +296%	6.68 (4.81–7.59)	3.80^b^ (2.43–5.69) −43%	2.31^b^ (1.84–3.25) −65%	2.16^b^ (1.98–2.63) −68%	2.47^a,b^ (2.05–2.84) +231%, −63%
Conn.D (mm^−3^)	Median (IQR) %diff	2 (1–3)	7^a^ (5–12) +261%	8^a^ (6–10) +300%	8^a^ (6–10) +287%	25 (14–44)	11^b^ (7–30) −55%	7^b^ (4–18) −74%	6^b^ (4–8) −76%	6^a^, ^b^ (4–7) +196%, −77%
Tb.Pf. (1/mm)	Median (IQR) %diff	13.0 (9.3–14.1)	5.2^a^ (3.7–6.4) −60%	5.3^a^ (3.7–6.7) −59%	5.3^a^ (4.1–6.8) −59%	−6.9 (−13.9 to 1.4)	5.8^b^ (4.7–7.3) −184%	6.2^b^ (5.1–7.9) −190%	6.1^b^ (5.5–8.2) −188%	5.1^a,b^ (3.1–5.6) −60%, −175%

Data were reported as the median, interquartile range (IQR), and percentage difference (%diff) with respect to the Met_VOI for each group. For the control group, comparison was reported against the lytic Met_VOI and blastic Met_VOI, respectively. “NS” represents not significant differences.

*BV/TV*, bone volume fraction; *Tb.Th.*, trabecular thickness; *SD_Tb.Th.*, standard deviation of the trabecular thickness; *Tb.Sp.*, trabecular separation; *Tb.N.*, trabecular number; *BS/TV*, bone surface density; *Conn.D*, connectivity density; *Tb.Pf.*, trabecular pattern factor

a Significantly different from the Met_VOI for lytic lesion at *p* < 0.05

b Significantly different from the Met_VOI for blastic lesion at *p* < 0.05

**Figure 5:**
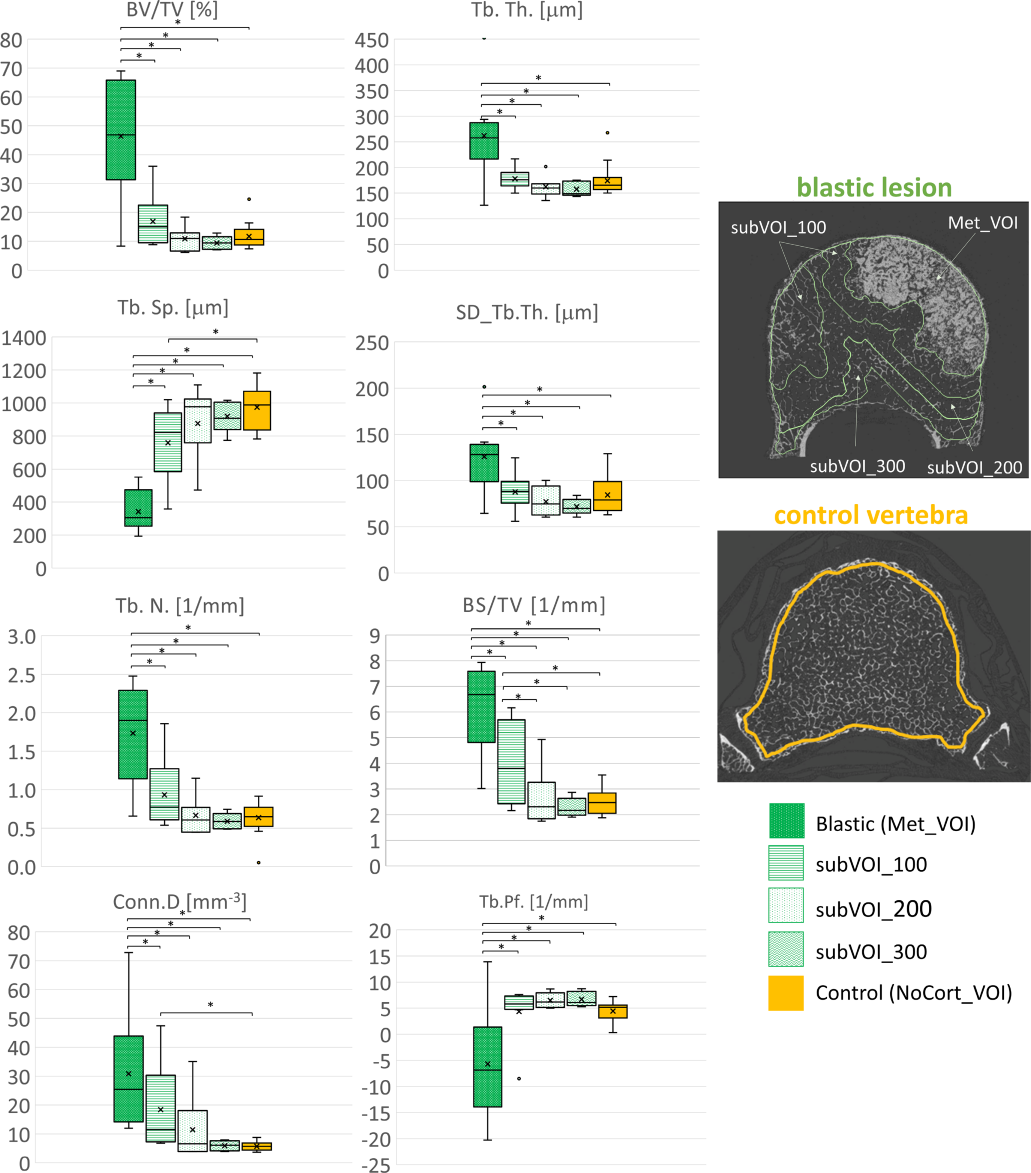
Box plot of the 3D microstructural parameters of the blastic metastases (*green*, *dots dark background*), of the trabecular bone in subVOI_100 (*green*, *horizontal line*), subVOI_200 (*green*, *dots white background*), and subVOI_300 (*green*, *zigzag line*), and the control vertebrae (*yellow*) in NoCort_VOI. The *box* is limited by the first and the third quartile. *Whiskers* represent the lowest and highest data points in the dataset excluding any outliers. The mean and median values over the group are represented by a *cross* and a *horizontal line*, respectively. Statistically significant differences between the metastatic groups and control group are highlighted with an *asterisk*.

In vertebral bodies with blastic metastases, most of the microstructural parameters resulted significantly different among the different VOIs: BV/TV (*F* = 20.3, *p* < 0.001, ICC = 0.0914), Tb.Th. (*F* = 7.67, *p* < 0.001, ICC = 0.00711), SD_Tb.Th. (*F* = 6.69, *p* < 0.001, ICC = 0.151), Tb.Sp. (*F* = 26.8, *p* < 0.001, ICC = 0.373), Tb.N. (*F* = 14.0, *p* < 0.001, ICC = 0.101), BS/TV (*F* = 17.8, *p* < 0.001, ICC = 0.0621), Conn.D (*F* = 6.73, *p* < 0.001, ICC = 0.00925), and Tb.Pf. (*F* = 8.09, *p* < 0.001, ICC = 0.0270). In particular, the blastic metastases (Met_VOI) (**Table 3**, **Figure 5**) exhibited higher values of BV/TV, Tb.Th., SD_Tb.Th., Tb.N., BS/TV and Conn.D (*P*<0.001) and lower values of Tb.Sp. and Tb.Pf. (*P*<0.001) than those measured in the subVOIs and the control vertebrae. All of the 3D microstructural parameters measured in the subVOIs of vertebrae with blastic metastases were not significantly different (*p* > 0.086). Only the BS/TV evaluated in subVOI_100 of blastic vertebrae differed from those of subVOI_200 (+64%; *p* = 0.027) and subVOI_300 (+76%; *p* = 0.014). Moreover, all of the 3D microstructural parameters measured in the subVOIs of vertebrae with blastic metastases were similar to those measured in the control vertebrae (*p* > 0.094). Only the Tb.Sp. (−17%; *p* = 0.009), BS/TV (+54%; *p* = 0.006), and Conn.D (+100%; *p* = 0.030) evaluated in subVOI_100 of blastic vertebrae differed from the control vertebrae (**Figure 5**).

## 4 Discussion

The aim of this study was to comprehensively assess the microstructural alterations in human vertebral bodies with lytic, blastic, and mixed metastases and compare them with vertebrae (controls) with no apparent observed lesion on the qCT from the same donors. These data were used to evaluate whether and to what extent the tissue surrounding the metastases was affected by the lesions.

The 3D microstructural parameters evaluated in the control vertebral bodies were in the same range of values reported in the literature obtained from healthy human thoracolumbar vertebrae, scanned with a voxel size from 6 to 82 μm (13, 16–28). These results confirmed that control vertebrae (without any metastatic features visible from the qCT scans) (36) from donors with spinal metastases had microstructural properties similar to those measured in healthy vertebrae from healthy donors.

Previous studies on entire vertebrae from rats (30) or on bone cores from human vertebrae (7, 22, 31) showed that lytic metastases degraded the trabecular bone by reducing the bone volume fraction, the trabecular number, and connectivity by eliminating rather than thinning the trabeculae with respect to healthy rat vertebrae or bone cores from healthy tissue from the same specimens. Moreover, lytic metastases were found to increase the separation between trabeculae in the vertebral body sections with respect to both blastic and mixed metastases (31). Contrary to what has been reported in the literature, the microstructural parameters measured in vertebrae with lytic metastases showed similar ranges to healthy vertebrae (13, 16–26). Indeed, the analysis of the entire vertebral body (NoCort_VOI) did not show any statistical differences of the vertebral bodies with lytic lesions when compared with the vertebral bodies without any lesion. These unexpected results are likely to be due to the relatively small size of the lytic lesions (the lytic metastasis volume ranged between 3% and 49% of the volume of the vertebral body, with an average lytic volume of 18%). The focal effect of lytic metastases was confirmed by the regions surrounding the lesion (Trab_VOI), which exhibited a bone microstructure similar to that of the control vertebral bodies from the same donors. These important findings highlighted for the first time that the tissue surrounding small lytic metastases is not affected by the lesions.

Vertebral bodies with blastic metastases exhibited a denser (+161% BV/TV, +27% Tb.Th., +99% Tb.N., and +107% BS/TV), more heterogeneous (+36% SD_Tb.Th.), less anisotropic (−13% DA), and more connected (+378% Conn.D and −144% Tb.Pf.) structure compared with the control vertebral bodies. These findings confirmed previous results on human cylindrical specimens extracted from blastic metastatic tissue and scanned using synchrotron radiation microCT (SR-μCT) (16, 21) or using microCT (7, 31). Indeed, in previous studies, vertebrae with blastic metastases exhibited larger BV/TV, Tb.Th., Tb.N., and Conn.D and lower DA and Tb.Pf. with respect to healthy vertebrae (13, 16–26). Despite the more isotropic arrangement of the tissue within the blastic regions suggesting a less optimised structure, vertebral bodies with blastic lesions were found to be stiffer and stronger than the vertebrae with lytic and mixed metastases (7, 31, 36, 47). This behaviour is mainly due to the denser tissue with respect to that of control vertebrae (16, 21, 31, 36, 47) and the frequent presence of diffuse sclerosis together with blastic metastases (16, 21).

Even in vertebrae with mixed metastases the microstructural parameters ranged significantly differently compared with from previously studied healthy vertebrae (13, 16–26). Moreover, the microstructural parameters in vertebrae with mixed metastases significantly differed from those measured in the control vertebrae in NoCort_VOI. Since the blastic tissue was significantly larger than the lytic one (+458%), the microstructural parameter measured in vertebrae with mixed metastases showed a similar trend to vertebral bodies with blastic lesions (**Figure 2**). As previously reported for bone cores from human vertebrae with mixed metastases (7), our findings showed that mixed metastases led to a denser (+230% BV/TV and +82% Tb.Th.), more heterogeneous (+91% SD_Tb.Th.), and less anisotropic (−14% DA) structure compared with the control vertebrae from the same donors. Similarly to vertebrae with blastic metastases, the thickening of the trabeculae and their higher heterogeneity in vertebrae with mixed lesions are likely to be associated with degenerative sclerosis of the bone rather than with bone metastases (16, 21). The 3D microstructural parameters of the vertebral bodies with mixed metastases were found to be different from those of the control vertebrae (**Table 2**) even in the tissue surrounding the lesions (Trab_VOI). The bone tissue was found to be denser (+112% BV/TV, +42% Tb.Th., and +112% Conn.D), more heterogeneous (+53% SD_Tb.Th.), and less isotropic (−17% DA) than that of the adjacent control vertebrae. It remains to be investigated whether this higher heterogeneity of the structure in vertebrae with mixed lesions compared with the other types of vertebrae is associated with higher or lower mechanical properties (7, 31, 36, 47, 48).

The LMM revealed that the morphological features in the vertebrae of each donor were correlated with the metastatic vertebrae (average ICC equal to 0.57); however, this correlation was reduced for the non-metastatic tissue (average ICC equal to 0.33), highlighting that the microstructure of the tissue outside the identified lesions is similar across the donors.

The local microstructural analysis performed within the volume of the metastatic lesion explained the alteration of the microstructural features previously observed in the whole vertebral body. The microstructural characterisation of the volume of the lytic and blastic lesions (Met_VOI) is in line with previous results reported for bone cores (7, 16, 21, 22). In vertebrae with lytic metastases, the BV/TV, Tb.Sp., Tb.N., BS/TV, Conn.D, and Tb.Pf. were different between the metastatic (Met_VOI) and the surrounding tissue (subVOI_100, subVOI_200, and subVOI_300) (**Table 3**). In turn, in most cases, the 3D microstructural parameters measured in the regions surrounding the lesion were found to be similar to those measured in the control vertebrae, highlighting the focal effect of the lesions.

The microstructure within blastic lesions was significantly different from that in the tissue surrounding the lesions (subVOI_100, subVOI_200, and subVOI_300) and in the control vertebrae for every investigated parameter. Moreover, most of the subVOIs surrounding the blastic lesions showed 3D microstructural parameters similar to those measured in the control vertebrae, highlighting that also blastic lesions, as lytic ones, alter the local microstructure of the vertebral body. Only in a few cases did blastic metastases also affect the tissue surrounding the lesion, with differences in Tb.Sp., BS/TV, and Conn.D, suggesting an increase of the number of connections in the trabecular network in the periphery of the lesion (21).

There are some limitations in this study. As the goal of this study was to evaluate the microstructure of the whole vertebral body affected by the lesions, a compromise had to be accepted between the image resolution (voxel size, 39um) and the size of the scanned region. A better spatial resolution could help in better identifying the interface between the metastatic lesion and the adjacent tissues, improving the segmentation. This is particularly critical for the vertebral bodies with mixed lesions with degenerative sclerotic tissue, but less critical for vertebral bodies with lytic and blastic metastases, which had focal lesion clearly distinguishable from the surrounding trabecular bone tissue. Nevertheless, the approach used followed the good practice guidelines (15) and enabled the evaluation of the 3D microstructural parameters on the whole vertebral body, highlighting the heterogeneity of the microstructure in these complex specimens. The second limitation is the sample size. In fact, the 50 vertebrae used in this work were harvested from 15 different donors of varied age and sex, affected by eight different types of primary tumours. Therefore, it was not possible to group the specimens with respect to age, sex, or tumour type in order to explore their effects on the bone microstructure. However, the spine segments were initially prepared including, for each metastatic vertebra, an adjacent control vertebra in order to avoid bias between the metastatic and control vertebrae introduced by age and sex and to clearly show the effects of the metastases on the bone microstructure.

In conclusion, this is the first study to evaluate the effect of metastatic lesions on the microstructure of the whole human vertebral body. Significant differences were observed within the metastatic vertebrae and the control vertebrae with no apparent observed lesion on the qCT. Furthermore, the focal effect of the lesion was highlighted by the differences observed in the volume of the metastases compared with the control vertebral bodies and with the tissue surrounding the lesions.

## Data Availability

The original contributions presented in the study are included in the article/**Supplementary Material**. Further inquiries can be directed to the corresponding author.
